# The limited role of women in small-scale fisheries in Mogadishu, Somalia; cultural norms, access to resources and educational attainment

**DOI:** 10.3389/fsoc.2026.1785217

**Published:** 2026-05-29

**Authors:** Abshir Abdisheekh Mohamed, Osman Elmi Omar, Abdinor Abukar Ahmed, Liban Abdisalam Mohamed, Imadudin Abdullahi Bilow

**Affiliations:** 1 Department of Marine Science, Somali National University, Mogadishu, Somalia; 2 Benaadir Research, Consultancy and Evaluation Centre, Mogadishu, Somalia

**Keywords:** access to resources, cultural norms, educational attainment, Mogadishu, small-scale fisheries, women’s participation

## Abstract

Women made vital yet often overlooked contributions to Mogadishu’s small-scale fisheries, particularly in post-harvest processing and trade. However, their participation remained constrained by socio-cultural norms, unequal access to productive resources, and limited educational attainment. This study examined the determinants of women’s participation in small-scale fisheries in Mogadishu by drawing on Social Role Theory, Access Theory, and Human Capital Theory. Cross-sectional survey data were collected from 80 women and analyzed using probit and logit regression models, along with marginal effects. The findings showed that fisheries experience, supportive social expectations, access to fishing equipment, and participation in fisheries training significantly increased women’s likelihood of participation. Cooperative membership and full household income contribution further strengthened these relationships. Overall, the results indicated that women’s participation was shaped by intersecting cultural, resource-related, and skills-based factors rather than by individual attributes alone. The study provided context-specific empirical evidence from Mogadishu and highlighted the need for gender-responsive policies that improve access to resources, strengthen training opportunities, support women’s cooperatives, and address restrictive norms in order to promote more inclusive fisheries governance in Somalia.

## 1 Introduction

Fisheries make significant contributions to the lives of millions of people worldwide as they serve as a source of food, nutrition, income, and tradition, and provide some of the most traded food commodities in the world (FOA, 2020). Small-scale fisheries usually take place in communities and are closely fused with local livelihoods (FOA, 2015). Many small-scale fisheries workers are self-employed and provide fishing resources for direct consumption within their households (FOA, 2020). Women are significant participants in the fishery sector, particularly in post-harvest and processing activities (FOA, 2020). Women contribute enormously to fisheries worldwide, especially in activities that require spending more time on land, such as manufacturing and fixing fishing gear, processing the catch, inspecting quality, commercializing the catch, and participating in conservation-related activities(Harper et al., 2013a; Kleiber et al., 2014). However, the definition of “fishery” often excludes these activities, hampering women’s access to rights, subsidies, and means of production (Kleiber et al., 2014).

Globally, according to the Food and Agriculture Organization (FAO), approximately 60 million people indirectly or directly work, either full or part time, in the primary sector of capture fisheries and aquaculture worldwide, and women constitute about 14% of the individuals directly involved in the fisheries and aquaculture sector (FOA, 2020). However, when fisheries are evaluated in their entirety, including processing and commercialization, women have been found to constitute half of the global fisheries workforce when post-production activities are included (FOA, 2020; World Bank, 2012). This wider view matters because women’s labor is often hidden in statistics that focus only on catching fish, even though women are active in processing, marketing, retailing, gleaning, and other shore-based activities that sustain fisheries households and local economies (Harper et al., 2013b; Kleiber et al., 2014).

The persistent marginalization of women in small-scale fisheries reflects the combined influence of structural, economic, and socio-cultural constraints that shape women’s agency, participation, and access to benefits. To explain this pattern, the study draws on three complementary theoretical perspectives. Social Role Theory explains how gendered expectations and culturally sanctioned divisions of labor assign women primarily to domestic and post-harvest roles, while male activity remains associated with fishing, mobility, and control over productive assets, a pattern that is especially relevant in Mogadishu where long-standing social norms continue to shape women’s involvement in fisheries (Eagly and Wood, 1987; Gawronski, 1995; Koenig et al., 2014). Human Capital Theory further suggests that limited schooling, training, and technical skills can restrict women’s productivity, employment opportunities, and ability to expand their role in fisheries, thereby making education and capacity-building central to participation outcomes (Becker, 1975; Blair, 2011; Deming, 2023). Access Theory complements these perspectives by showing how exclusion from boats, landing sites, finance, markets, and social networks limits women’s ability to benefit meaningfully from fisheries resources, even when they are physically present in the sector (Peluso and Ribot, 2020; Ribot and Peluso, 2003). Taken together, these theories show that women’s limited participation is not incidental but is produced through the interaction of restricted access, limited human capital, and socially reinforced gender roles.

In Africa, Eastern Africa had the highest women participation rate at 26% (±11%), whereas Northern Africa had the lowest at 2% (±1%) (Harper et al., 2013b). A study from Tanzania shows that 44% of women expressed discomfort with working in the sector due to challenges such as low income, marginalization, and environmental insecurities (Rwechungura, 2024a). Another study conducted in the Dagaa fishery value chain in Lake Victoria, Kenya, revealed that women’s involvement is limited by disparities in the ownership of boats and fishing gear, together with limited access to finance and fisheries resources (Peluso and Ribot, 2020). These findings show that women’s participation is not simply a matter of willingness, but also a result of structural and social conditions that shape who can enter, remain in, and benefit from fisheries.

In Somalia, fisheries are almost entirely small-scale, relying on traditional gear, small vessels, and artisanal landing sites along the coast. Small-scale fisheries represent a vital livelihood source, yet gendered divisions of labor remain pronounced. Studies conducted at Mogadishu’s major landing sites demonstrate that fishing is usually done by men in the capital and is considered male work in Somali society (Hassan and Hossain, 2023). The same study showed that women working at the Liido, Urubo, and Abaydhahn landing sites are mainly traders who buy fish from the beach and then resell it in town (Hassan and Hossain, 2023). Similar patterns have been observed in broader coastal assessments, where women are mostly responsible for skilled and time-consuming onshore tasks, such as making and mending nets, baskets, and pots; processing and marketing catches; and providing services to boats, such as selling fuel or renting refrigerators and boats to fishermen (CISP, 2018). Women are also wholesalers and retailers. As wholesalers, they purchase fish in bulk from fishers or cooperative societies and sell it to retailers. As retailers, women purchase fish from wholesalers and transport it to their selling points (CISP, 2018).

A common stereotype is that women have no capacity to perform difficult physical tasks such as fish catching, hunting, repairing boats, tools, and gears. Some men do not appreciate their wives working in fisheries because they consider the sector not to be very lucrative. However, interviewed women believe that if given knowledge and opportunities, and if allowed to have a share in sales and market prices, they would know how to exploit more business opportunities (CISP, 2018). This is important for Mogadishu because the city is a coastal urban center where women are already active around landing sites, markets, and processing chains, yet their roles are often limited to low-value activities. The problem is therefore not the absence of women in fisheries, but the limited recognition of their contribution, the restricted access to productive resources, and the persistence of social norms that confine them to less visible and less rewarded roles.

Although the literature on women and fisheries is expanding internationally, there remains a clear gap in peer-reviewed evidence focused on Somalia. In particular, there is no systematic empirical evidence examining how cultural norms, access to resources, and educational attainment interact to shape women’s participation in small-scale fisheries in Mogadishu. Much of the existing discussion on Somalia remains descriptive and policy-oriented rather than analytically grounded, limiting the ability to generate context-specific insights. This gap is especially critical in Mogadishu, where small-scale fisheries constitute an important livelihood source, yet women’s roles remain largely invisible, under-documented, and insufficiently understood. As a result, national planning frameworks risk underestimating women’s economic contributions while overlooking the structural and socio-cultural barriers that constrain their progression into more productive and remunerative roles within the sector.

This evidence gap is further compounded by Somalia’s current phase of economic recovery and institutional transformation. The country has recently benefited from debt relief and launched the National Transformation Plan (NTP) 2025–2029, which positions fisheries as a strategic sector for national growth and identifies women’s empowerment as central to achieving inclusive and equitable development (NTP, 2025). In alignment with this agenda, initiatives such as the Women in Marine Sector (WiMS) program, launched in 2019 in coordination with UNISOM, seek to enhance women’s access to fisheries resources and participation in decision-making processes. However, the continued reliance on targeted interventions underscores the persistence of gender disparities within the sector and highlights the urgent need for rigorous, location-specific empirical evidence to inform effective and sustainable policy responses.

In response to this gap, the present study examines the determinants of women’s participation in small-scale fisheries in Mogadishu, with particular attention to cultural norms, access to resources, and educational attainment. By focusing on these interrelated dimensions, the study seeks to provide a clearer understanding of the factors that shape women’s roles in the sector and to situate Mogadishu within the broader discourse on gender and fisheries. In this respect, the study makes three important contributions. First, it generates much-needed empirical evidence from a context that has received limited analytical attention, thereby strengthening the scholarly understanding of women’s participation in Somalia’s fisheries sector. Second, it contributes theoretically by bringing together Social Role Theory, Human Capital Theory, and Access Theory within a coherent explanatory framework. Third, it offers policy-relevant insights that may inform more inclusive and gender-responsive interventions aimed at improving women’s access to productive resources, strengthening their participation, and addressing the socio-cultural barriers that continue to constrain their engagement in fisheries.

## 2 Methodology

This study employed a quantitative cross-sectional research design to examine the factors influencing women’s limited participation in small-scale fisheries in Mogadishu, Somalia. Primary data were collected using an interviewer-administered structured questionnaire to ensure clarity and accuracy of responses, particularly in the context of varying literacy levels among participants. The questionnaire was initially prepared in English, translated into Somali for field administration, and subsequently back-translated into English to ensure consistency and reliability of meaning. Data were collected from three major fish markets in Mogadishu—Lido, Hamarweyne, and Wadajir—which represent the primary operational hubs for small-scale fisheries activities in the city. Due to the absence of a centralized database in Somalia’s fisheries sector regarding the number of women working in the fisheries sector of Banadir Region, probabilistic sampling was not feasible. A purposive sampling approach was therefore applied over 6 months (August–December 2025) through direct field visits and cross-sectioning data collection. This process resulted in a final sample of 80 women, representing all eligible participants available during the data collection period. By engaging all accessible women rather than selecting a predetermined number, the study ensured a more complete and authentic reflection of the female workforce in Mogadishu’s small-scale fisheries.

The study conceptualizes women’s participation in small-scale fisheries as a multidimensional construct, measured using multiple indicators derived from the questionnaire. Rather than aggregating these indicators into a composite index, each item capturing participation was treated as a separate dependent variable to reflect different dimensions of engagement in fisheries activities. Specifically, participation was measured using indicators such as involvement in fishing activities, level of engagement (full-time or part-time), volume of fish handled, cooperative membership, and willingness to expand participation if constraints are reduced. These variables were measured using binary and categorical scales, depending on the nature of each question. Independent variables were grouped into three categories: Cultural norms: These include community encouragement, cultural restrictions, decision-making inclusion, household responsibilities, stigma, and male household support. Access to resources: These include access to fishing equipment, funding sources, market access, equality in resource access, and etc. Educational attainment: This includes level of education, participation in fisheries-related training, perceived impact of education, and equality of educational opportunities. All items were measured as binary variables (Yes = 1, No = 0). In addition, selected demographic characteristics, such as fisheries experience, were included in the analysis as control variables.

Given that the indicators of women’s participation were measured as binary, logistic and probit regression models were employed to estimate the relationship between explanatory variables and each participation outcome. Each participation indicator was modeled separately as a dependent variable to capture distinct dimensions of women’s involvement in small-scale fisheries. This approach allows for a more nuanced understanding of how cultural norms, access to resources, and educational attainment influence different aspects of participation. A Linear Probability Model (LPM) was also estimated as a baseline specification for comparison. The use of multiple estimation techniques enhances the robustness of the findings and allows for consistency checks across models. Empirical framework for the study was specified using three complementary models. First, the Linear Probability Model (LPM) was specified as follows in Equation 1:


$${\mathrm{WP}}_{i}\;=\;\;\beta_{0}+\beta_{1}{\mathrm{CN}}_{i}+\beta_{2}{\mathrm{AR}}_{i}+\beta_{3}{\mathrm{EA}}_{i}+E_{i}$$ (1)

where ${\mathrm{WP}}_{i}$ is Women participation. ${\mathrm{CN}}_{i}$ is cultural norms (e.g., social expectation and gender role). ${\mathrm{AR}}_{i}$ are access to resource factors, such as access to finance, access to equipment, access to markets and access to fisheries. ${\mathrm{EA}}_{i}$ : are educational attainment factors, including schooling, training and skill. $E_{i}$ : is the error term.

Second, the Logit model was expressed as in Equation 2:


$$P(\mathrm{WP}=1)=\frac{\exp(\beta_{0}+\beta_{1}CN_{i}+\beta_{2}AR_{i}+\beta_{3}EA_{i})}{1+\exp(\beta_{0}+\beta_{1}CN_{i}+\beta_{2}\mathrm{AR}+\beta_{3}EA_{i})}$$ (2)

Third, the probit model was expressed as in Equation 3:


$$P(\mathrm{WP}=1)=\int_{-\infty }^{\left(\beta_{0}+\beta_{1}{\mathrm{CN}}_{i}+\beta_{2}{\mathrm{AR}}_{i}+\beta_{2}{\mathrm{EA}}_{i}\right)}\emptyset (z)\mathrm{dz}$$ (3)

Data analysis was conducted using STATA version 17. Prior to analysis, the dataset was cleaned by checking for missing values, inconsistencies, and duplicates, and no significant missing data were identified. Multicollinearity among independent variables was assessed using the Variance Inflation Factor (VIF), with all values falling within acceptable thresholds, indicating no serious multicollinearity concerns. The reliability of the measurement items was evaluated using factor loadings and Cronbach’s alpha to assess internal consistency. The results of these diagnostic tests are presented and discussed in the following section. Ethical approval was obtained from the Institutional Review Board (IRB) of Somali National University (SNU) (Ref: JUS/FSC/00071/25, dated July 20, 2025). Informed consent was obtained from all participants prior to data collection, and anonymity and confidentiality were strictly maintained throughout the study.

## 3 Results and discussion

### 3.1 Results

Table 1 presents the results of the reliability and validity assessment of the measurement items. The findings indicate that all constructs demonstrate acceptable levels of internal consistency and convergent validity. Factor loadings for all items are above commonly accepted thresholds, indicating strong associations between observed variables and their respective constructs. The squared multiple correlations (SMC) further show that the items explain a substantial proportion of variance in their underlying factors. In addition, Cronbach’s alpha values for Women’s Participation, Cultural Norms, Access to Resources, and Educational Attainment exceed recommended levels, confirming good internal consistency. Overall, these results suggest that the measurement instruments are reliable and suitable for subsequent multivariate analysis.

**Table 1:** Factor loading, square multiple correlation, and Cronbach’s alpha.

Constructs	Items	Factor loading	SMC	Alpha
Women’s participation	WP1	0.9232	0.8522	0.8114
	WP2	0.7716	0.5953	
	WP3	0.7813	0.6104	
	WP4	0.7369	0.5430	
	WP5	0.6330	0.4006	
Cultural norms	CN1	0.8242	0.6793	0.8342
	CN2	0.7163	0.5130	
	CN3	0.7540	0.5685	
	CN4	0.8513	0.7247	
	CN5	0.8210	0.6740	
	CN6	0.7416	0.5499	
Access to resources	AR1	0.6487	0.4208	0.7439
	AR2	0.7167	0.5136	
	AR3	0.7625	0.5814	
	AR4	0.7718	0.5956	
	AR5	0.8217	0.6751	
	AR6	0.7819	0.6113	
	AR7	0.7322	0.5361	
Educational attainment	EA1	0.6418	0.4119	0.7263
	EA2	0.9320	0.8686	
	EA3	0.9346	0.8734	
	EA4	0.8121	0.6595	

In addition, Table 2 reports the variance inflation factor (VIF) and tolerance values used to assess multicollinearity among the explanatory variables. All variables exhibit low VIF values and high tolerance levels, indicating that multicollinearity is not a concern in the regression models. This confirms that the independent variables are sufficiently distinct and that the estimated regression coefficients are reliable and stable.

**Table 2:** Variance inflation factor and tolerance for multicollinearity tests.

Variables	(1)	(2)	(3)	(4)	(5)	(6)	VIF	Tolerance
Women’s participation	1						1.08	0.921793
Cultural norms	0.128	1					1.06	0.946849
Access to resources	−0.149	−0.025	1				1.05	0.951983
Educational attainment	0.163	0.078	−0.134	1			1.03	0.970628
Experience	0.078	0.056	0.152	−0.089	1		1.01	0.989193

Furthermore Table 3 presents the demographic characteristics of women engaged in small-scale fisheries in Mogadishu. The result shows that the majority of respondents are aged 40–50 years (46.25%), indicating that participation is concentrated among middle-aged women. Most respondents are married (53.75%), suggesting a strong link between fisheries activities and household responsibilities. In terms of experience, the largest group has more than 10 years of experience (27.50%), reflecting sustained engagement in the sector. A significant proportion of women operate at Liido landing site (63.75%), highlighting its dominance as a key fisheries hub. Household sizes are generally large, with both 7–10 members and more than 10 members (each 41.25%) being the most common, indicating high dependency levels. The majority of respondents fully contribute to household income (66.25%), emphasizing the economic importance of fisheries for women. However, income levels remain low, with most respondents earning USD 100–300 per month (60.00%), reflecting limited access to higher-value opportunities. Overall, these findings suggest that women’s participation is driven by experience, household responsibilities, and economic necessity within constrained structural conditions.

**Table 3:** Demographic characteristics of women engaged in small-scale fisheries in Mogadishu.

Variable	Category	Frequency (N)	Percent (%)
Age	18–28 years	1	1.25
	29–39 years	30	37.50
	40–50 years	37	46.25
	51 + years	12	15.00
Marital status	Divorced	17	21.25
	Married	43	53.75
	Single	1	1.25
	Widowed	19	23.75
Experience	<1 year	2	2.50
	1–3 years	19 h	23.75
	4–6 years	20	25.00
	7–10 years	17	21.25
	>10 years	22	27.50
Landing site	Liido	51	63.75
	Xamarweyne	29	36.25
	Wadajir		
Household size	1–3	2	2.50
	4–6	12	15.00
	7–10	33	41.25
	>10	33	41.25
Contribution to household income	Fully	53	66.25
	Partly	27	33.75
Monthly income (USD)	100–300	48	60.00
	301–500	13	16.25
	501–700	12	15.00
	>700	7	8.75
Total		80	100

Moreover, Table 4 presents a cross-tabulation analysis of factors influencing women’s participation in small-scale fisheries in Mogadishu. It highlights statistically significant associations between women’s participation in small-scale fisheries and key explanatory factors. Participation surges with greater fisheries experience, and is higher among women who receive family support from men. In addition, access to fishing equipment and formal fisheries training are both strongly related with higher participation levels. These results indicate that women’s participation is formed by experience, supportive social conditions, access to productive resources, and skills development.

**Table 4:** Cross-tabulation analysis of the study factors.

Study factors	Role of women in small-scale fisheries			
	No	Yes	Total	Chi square prob
How long have you been working in this sector?				
1–3 years	15	4	19	0.000
4–6 years	19	1	20	
7–10 years	8	9	17	
Less than 1 year	2	0	2	
above 10 years	3	19	22	
Do men in your household support your fisheries activities?				
No	22	4	26	0.001
Yes	25	29	54	
Do you own or have access to fishing equipment (boats, nets, machines, gear)?				
No	45	13	58	0.000
Yes	2	20	22	
Have you ever received formal training related to fisheries?				
No	37	8	45	0.000
Yes	10	25	35	
Total	47	33	80	

The study additionally used a probit regression model to estimate key determinants of women’s participation among SSFs in Mogadishu. Table 5 presents the consolidated regression results examining the determinants of women’s limited participation in small-scale fisheries in Mogadishu across linear probability, logit, and marginal effects specifications. The results consistently indicate that fisheries experience, social expectations, access to fishing equipment, and fisheries training are statistically significant predictors of women’s participation outcomes. Across all model specifications, fisheries experience exhibits a positive and significant association, suggesting that longer engagement in fisheries-related activities increases women’s likelihood of active participation. Social expectations also demonstrate a robust positive effect, highlighting the importance of supportive cultural and social norms in shaping women’s roles within the fisheries sector. Similarly, access to fishing equipment emerges as one of the strongest determinants, underscoring the critical role of material resources in enabling meaningful participation. Fisheries training is likewise significant, indicating that skill acquisition and formal capacity-building opportunities enhance women’s involvement in small-scale fisheries.

**Table 5:** Determinants of women’s limited participation in SSFs: regression results.

Predictors	Linear probability model		Logit model (odds ratios)		Marginal effects (dy/dx)	
	Model 1	Model 2	Model 3	Model 4	ME 1	ME 2
Fisheries experience	0.106*** (0.031)	0.071** (0.032)	0.698*** (0.238)	0.509* (0.267)	0.209*** (0.043)	0.204*** (0.046)
Social expectation	0.197** (0.090)	0.216** (0.085)	1.439** (0.711)	1.703** (0.781)	0.380*** (0.141)	0.302** (0.153)
Equipment access	0.428*** (0.111)	0.353** (0.107)	2.810*** (0.979)	2.602** (1.013)	0.625*** (0.206)	0.667*** (0.214)
Fisheries training	—	0.283*** (0.090)	—	1.969*** (0.747)	—	0.307** (0.155)
Constant	−0.763*** (0.167)	−1.010*** (0.176)	−8.226*** (1.908)	−10.750*** (2.364)	—	—
						
Observations	80	80	80	80	80	80
Pseudo *R*^2^	—	—	0.434	0.505	—	—

***p < 0.01, **p < 0.05, *p < 0.1.

Additionally, Table 6 reports logit estimates of women’s participation in small-scale fisheries disaggregated by cooperative membership status and household contribution level. The results reveal clear heterogeneity across groups. Among women who belong to cooperatives, fisheries experience has a strong and statistically significant positive effect on participation, indicating that accumulated experience translates into greater engagement when women operate within organized structures. Social expectations are also significant for cooperative members, suggesting that supportive social and cultural norms reinforce participation in formal fisheries groups. Access to fishing equipment emerges as a particularly strong determinant within cooperatives, highlighting the importance of material resources in enabling active involvement. By contrast, fisheries training does not exhibit a statistically significant effect for cooperative members.

**Table 6:** Women’s participation in small-SSFs: logit estimates by cooperative membership and household contribution.

Variables	Cooperative membership		Household contribution:	
	No	Yes	Partial	Full
Fisheries experience	0.38 (0.44)	1.063*** (0.28)	0.448 (1.516)	1.055* (0.632)
Social expectation	1.369 (1.264)	1.94** (0.934)	−0.623 (0.384)	−0.261 (0.18)
Equipment access	—	4.103** (1.653)	0.701 (1.114)	1.095* (0.574)
Fisheries training	—	0.36 (0.93)	—	0.717* (0.376)
Constant	−4.743* (2.608)	−6.559*** (2.072)	1.326 (3.084)	−2.463 (2.772)
Observations	24	56	24	270
Pseudo *R*^2^	0.092	0.383	0.237	0.134

***p < 0.01, **p < 0.05, *p < 0.1.

Furthermore, when participation is examined by household contribution status, the findings show that women who fully contribute to household income are more responsive to fisheries experience, access to equipment, and fisheries training, all of which positively and significantly influence participation. In contrast, for women who contribute only partially to household income, none of the explanatory variables demonstrate statistically significant effects. These patterns indicate that both organisational membership and economic responsibility within the household condition the extent to which experience, resources, and training translate into meaningful participation in small-scale fisheries.

### 3.2 Discussion

Our findings reinforce and extend insights from the literature. Consistent with Social Role Theory, traditional gender expectations were strongly associated with women’s participation: where norms discouraged women’s fishing, engagement was low. This mirrors broad evidence that patriarchal social structures and stereotypes marginalize women in fisheries management (e.g., communities where women’s opinions are undervalued (Chambon et al., 2024). The Somali context thus follows the global pattern of constrained roles for women in SSF. Importantly, our results empirically confirm that cultural attitudes materially impact participation decisions.

We also found that better access to fishing equipment and resources significantly increased women’s participation. This aligns with Access Theory: women who can secure capital for boats, nets, or gear are more able to fish. WorldFish and FAO have documented that women often lack such assets (FAO, 2015; WorldFish, 2020). By showing equipment access matters here, we add quantitative support to these observations. It suggests that practical interventions to provide women with tools or financing could help overcome structural disadvantages.

Similarly, fisheries training and experience were significant positive predictors of participation. This outcome is in line with Human Capital Theory: as women acquire specific skills and knowledge, they become more capable participants. Though some general surveys have found formal education alone may not suffice (Adisa et al., 2025; Rwechungura, 2024b), our analysis underscores the value of context-relevant training. Women who received fisheries training presumably gained both technical know-how and confidence, making them more likely to engage. Likewise, accumulated experience (years of involvement or informal learning) enhanced participation, indicating that skill accumulation over time matters. Our study thus highlights the importance of capacity-building programs for women in fisheries.

An important nuance from our results is the moderating role of cooperative membership. Women belonging to fishing cooperatives showed stronger participation effects from training and equipment. This suggests that social capital and collective organization amplify opportunities – a finding consistent with development literature on women’s groups (FAO, 2015). In practice, cooperatives may provide information-sharing, pooled resources, and a platform to assert women’s interests. By showing this interaction, our work contributes a new perspective: it is not only individual factors, but also the social context (membership) that enables women to leverage those factors effectively.

Conversely, higher household contribution levels tended to dampen women’s participation. This echoes the literature that unpaid domestic burdens limit women’s economic engagement (NOAA, 2020). In Mogadishu, women with heavier household duties had less time or flexibility to fish, even if they had access or skills. This finding reinforces Social Role Theory’s point that women’s caregiving roles constrain their work outside the home. It suggests any effort to promote women’s fisheries involvement must consider their unpaid workload.

## 4 Conclusion

This study finds that women’s participation in Mogadishu’s small-scale fisheries is shaped by interlocking social and economic factors. Traditional social expectations rooted in patriarchal norms act as a significant barrier, while limited access to fishing equipment, training, and financial tools further restrain women’s involvement. In contrast, women who gain access to these resources—particularly through fisheries training and accumulated experience—are more likely to participate, reflecting the relevance of Human Capital and Access Theories. Cooperative membership further strengthens these effects, while heavy domestic responsibilities reduce participation. These findings align with global evidence that gender norms and inequitable access to assets remain key constraints.

Importantly, the limited role of women in Mogadishu’s fisheries is not fixed but results from modifiable structural and normative barriers. Based on these findings, the study puts forward several key recommendations. Community engagement efforts are needed to challenge restrictive gender norms, while improved access to essential fishing equipment and financing—through microcredit or shared ownership—can reduce material barriers. Expanding fisheries-specific training programs will strengthen women’s technical capacity and confidence. Supporting the development of women-led cooperatives can enhance collective access to resources and decision-making platforms. Additionally, addressing domestic workload through flexible schedules or childcare options can enable women to engage more fully in fisheries activities. Together, these recommendations offer practical, evidence-based strategies to promote gender-equitable participation in Somalia’s small-scale fisheries.

## Data Availability

The raw data supporting the conclusions of this article will be made available by the authors, without undue reservation.
